# Anterior cruciate ligament deficiency versus intactness for outcomes in patients after unicompartmental knee arthroplasty: a systematic review and meta-analysis

**DOI:** 10.3389/fbioe.2022.890118

**Published:** 2022-08-23

**Authors:** Weiming Guo, Teng Wan, Haifeng Tan, Gang Fan, Xiaoyu Gao, Pan Liu, Changqing Jiang

**Affiliations:** ^1^ Department of Sports Medicine, Huazhong University of Science and Technology Union Shenzhen Hospital, The 6th Affiliated Hospital of Shenzhen University Health Science Center, Shenzhen, China; ^2^ Hengyang Medical College, University of South China, Hengyang, Hunan, China

**Keywords:** anterior cruciate ligament, surgery, meta, unicompartmental arthroplasty, knee

## Abstract

**Objective:** The unicondylar knee arthroplasty (UKA) procedure is primarily indicated for osteoarthritis of the knee. Anterior cruciate ligament (ACL) defects have long been considered a contraindication to UKA. However, recent clinical studies have found that ACL defects do not affect postoperative outcomes in UKA. To elucidate whether ACL defects affect postoperative outcomes in UKA, we performed a systematic review and Meta-analysis of observational cohort studies comparing the effects of ACL defects and intactness on surgical outcomes in UKA.

**Methods:** In this study, we used “Anterior Cruciate Ligament”, “Anterior Cruciate Ligament Injuries” and “Arthroplasty, Replacement, Knee” as the subject terms according to PICOS principles. These subject terms and the corresponding free texts were used to conduct a systematic search in the three major databases PubMed, Embase and Cochrane on December 9, 2021. The main study variables included age, gender, region, definition of ACL defect and diagnosed diseases. The study used a random effect model to pool the effect of 95% CIs. To explore the sources of heterogeneity and to test the stability of the results, a sensitivity analysis was performed.

**Results:** The systematic review found no significant differences in postoperative clinical outcomes in the elderly population when unicondylar replacement was performed in the setting of multiple factors such as injury, defects, longitudinal tear, and synovial bursa injury defined as ACL deficiency. The primary clinical outcomes included postoperative revision, Tegner activity score, and Oxford Knee Score (OKS). After statistical meta-analysis, postoperative outcomes such as postoperative revision (OR, 1.174; 95% CIs, 0.758–1.817) and Tegner activity score (OR, -0.084; 95% CIs, -0.320–0.151) were not statistically different.

**Conclusion:** There was no difference in postoperative revision rates and functional outcomes such as Tegner activity score between the ACL-deficient group compared with the ACL-intact group. For the present results, it is not advisable to consider ACL deficiency as a contraindication of UKA.

## Introduction

Osteoarthritis is currently the most common type of arthritis in the world and is one of the leading causes of pain, disability and increased socioeconomic costs worldwide (Bijlsma et al., 2011; Glyn-Jones et al., 2015). The pathology is characterized by degenerative changes in the bones, cartilage, menisci, ligaments and synovial tissues of the joints, and patients usually suffer from irregular chronic pain, which seriously affects their quality of life (Braun and Gold, 2012; Goldring and Goldring, 2016). The prevalence of knee osteoarthritis appears to be higher compared to other types of osteoarthritis (Bliddal and Christensen, 2009). Current research suggests that the rising incidence of knee osteoarthritis in the population is closely related to the aging of the population and the obesity epidemic (Bijlsma et al., 2011; Heidari, 2011). UKA is now a common treatment modality for many patients with knee osteoarthritis (Wilson et al., 2019). Compared to total knee arthroplasty (TKA), it has the advantages of less injury and faster recovery, but a higher revision rate (Murray and Parkinson, 2018). ACL rupture and injury are often caused by degenerative changes in the knee joint and are also likely to occur in young and active individuals (Louboutin et al., 2009). In addition, drastic biomechanical changes in the knee joint during sports and accidents can also lead to ACL (Larwa et al., 2021). A ruptured and defective ACL will result in anterior tibial translation and abnormal shearing forces to the posterior medial aspect of the knee (Filbay and Grindem, 2019). Further progression may also lead to degenerative tears of the posterior horn of the medial meniscus, thereby affecting joint stability (Frobell et al., 2010).

The current study shows that knee osteoarthritis and ACL deficits can be causally linked across age groups, which makes the two symptoms often appear together, especially after ACL damage, and patients have a significantly higher incidence of knee osteoarthritis (Friel and Chu, 2013; Mancuso et al., 2016; Cinque et al., 2018). Combined ACL reconstruction and UKA is one of the accepted treatment modalities for ACL defects combined with osteoarthritis. This treatment modality has performed better for younger patients with a mean of 2 years of follow-up, but longer-term observational studies are lacking (Volpin et al., 2018; Tecame et al., 2019). It is also of concern that ligament reconstruction, while it may help improve joint stability and functional prognosis, may also mean that TKA is more likely to be performed later than in the general population (Leroux et al., 2014). And as the need for TKA continues to increase in youngers, ACL reconstruction has been found to increase the risk of reoperation for TKA due to the need to remove the implant (Leroux et al., 2014; Watters et al., 2017). This suggests that ACL reconstruction combined with UKA treatment may also have long-term risks. In contrast, resection of the ACL has been found to have no impact on clinical outcomes such as maximum knee extension or overall limb alignment for TKA surgery (Hoogeslag et al., 2019). The scope of applicability of UKA needs to be further evaluated. Past studies have shown a clear association between ACL defects and failure of UKA surgery, which may be due to aseptic loosening of the tibial prosthesis in the early postoperative period (Goodfellow et al., 1988; Kozinn and Scott, 1989). Additionally, clinical studies have shown an increased failure rate of both fixed-axis and mobile-axis UKA procedures for ACL defects (Deschamps and Lapeyre, 1987; Goodfellow et al., 1988). Therefore, ACL defects or injuries have been considered a contraindication to UKA. However, given the advantages of UKA over TKA surgery, such as more bone reserves, less surgical injury, and faster recovery, patients with ACL defects still take this procedure (Price et al., 2001). In 2004, a study suggested that UKA was indicated in the absence of a history of knee instability in the setting of ACL deficiency (Engh and Ammeen, 2004). This view was supported by a clinical study in 2014 (Engh and Ammeen, 2014). A retrospective study highlighted that ACL deficiency was not a contraindication to UKA and that fixed-axis lateral UKA had been successful in patients with ACL deficiency (Plancher et al., 2014). A study in 2019 showed no significant differences in kinetic and kinematic outcomes between conventional UKA and ACL-deficient UKA (Suter et al., 2019). Notably, several post-UKA follow-up surveys have shown no significant difference in mean 3- or 5-years follow-up prosthesis survival rates between patients with ACL defects and those with intact ACLs (Boissonneault et al., 2013; Engh and Ammeen, 2014; Kikuchi et al., 2021). Recent studies have revealed no difference in postoperative revision and functional scores between ACL-deficient and intact patients undergoing UKA (Kikuchi et al., 2021; Plancher et al., 2021). This suggests that ACL defects may not be related to the loosening of the prosthesis after UKA surgery and that the suitability of ACL-deficient patients for UKA treatment remains controversial.

This study will perform a meta-analysis and systematic review of the impact of ACL defects and ACL integrity on clinical outcomes such as postoperative revision, Tegner activity score and OKS in patients. This study will explore whether ACL defects are contraindication to UKA, which will inform clinicians’ choice of surgical approach in the case of patients with ACL defects from an evidence-based medicine perspective.

## Methods

Results are reported using Preferred Reporting Items for Systematic Reviews and Meta-Analyses 2020 (PRISMA 2020) (Page et al., 2021).

### Search strategy

A systematic search of PubMed, Embase, and the Cochrane Library was conducted up to 12/09/2021. All medical subject headings and corresponding free texts were taken from the “Mesh Database”. Published research on other related topics was used to ensure the comprehensiveness and rationality of the terms used. The medical subject heading terms were used including “Anterior Cruciate Ligament” “Anterior Cruciate Ligament Injuries” “Arthroplasty, Replacement, Knee”. In addition, the reference lists of retrieved papers and reviews related to the impact of ACL status on UKA were screened to try to avoid possible omissions. Corresponding authors were not contacted for additional data. There were no language restrictions for the literature search (Supplementary Appendix S1).

### Study selection

Each of the 2 authors independently screened the articles and cross-checked the finalized ones. In case of disagreement on the inclusion and exclusion of a few articles, a third author made the final decision. In the first stage, we screened titles, and then in the second stage, we performed the abstract and full-text screening. Studies meeting the following criteria were included: (Glyn-Jones et al., 2015): study design: retrospective observational studies; (Bijlsma et al., 2011); participants: patients who underwent UKA surgery, with and those without ACL defects, mainly including fragile and broken ACL, tears, non-functionality, complete absence and synovial damage; (Braun and Gold, 2012); Outcomes: Postoperative revision due to prosthetic loosening, imaging findings of transilluminated bands, and wear of the lateral osteoarthrosis, Tegner activity score, and OKS, for which odds ratio (OR) or standard mean difference (SMD), and the corresponding 95% confidence intervals (CIs) were provided as the effect measures. Studies that did not compare ACL deficiencies with complete studies, non-original articles such as reviews, duplicate cohorts, and studies that did not report primary outcomes were excluded.

### Data extraction and quality assessment

Two authors independently extracted the following information from each study: 1) study characteristics, authors, year of publication, and study design; 2) study population characteristics, such as total number of knees with UKA, age, and sex ratio of participants in each group; 3) intervention characteristics, such as definition of ACL deficits; 4) clinical outcomes such as postoperative revision, Tegner activity score, and OKS and their mean follow-up time. The final decision on disagreement was made by a third senior author. Quality scores were derived by applying the Newcastle Ottawa Scale. A score of less than or equal to 7 is considered a low risk of bias (Supplementary Appendix Table S3).

### Statistical analysis

The primary outcome analyzed was postoperative revision, defined as conversion from UKA to TKA or otherwise. Secondary outcomes included Tegner activity score and OKS. we extracted adjusted effect estimates. During continuous data extraction, some studies were reported as medians, which we converted to mean and standard deviation form by arithmetic (Wan et al., 2014; Luo et al., 2018). Stata software (15.1 version) was used to perform the data analysis. Meta-analysis of outcomes reported by three or more studies was performed by using a random effects model. Binary variables were reported with odds ratio (OR) and continuous variables were reported with standard mean difference (SMD). We applied an algorithm to combine SMDs for multiple ACL deficiency-related subgroups (Cumpston et al., 2019). ORs converted to logs were combined using the generalized inverse variance method and random effects models and effect estimates and their 95% confidence intervals are reported. I^2^ values above 50% indicate significant heterogeneity (Higgins et al., 2003). Begg’s test was not performed because the number of included studies was less than 10.

## Results

### Search results

**FIGURE 1:**
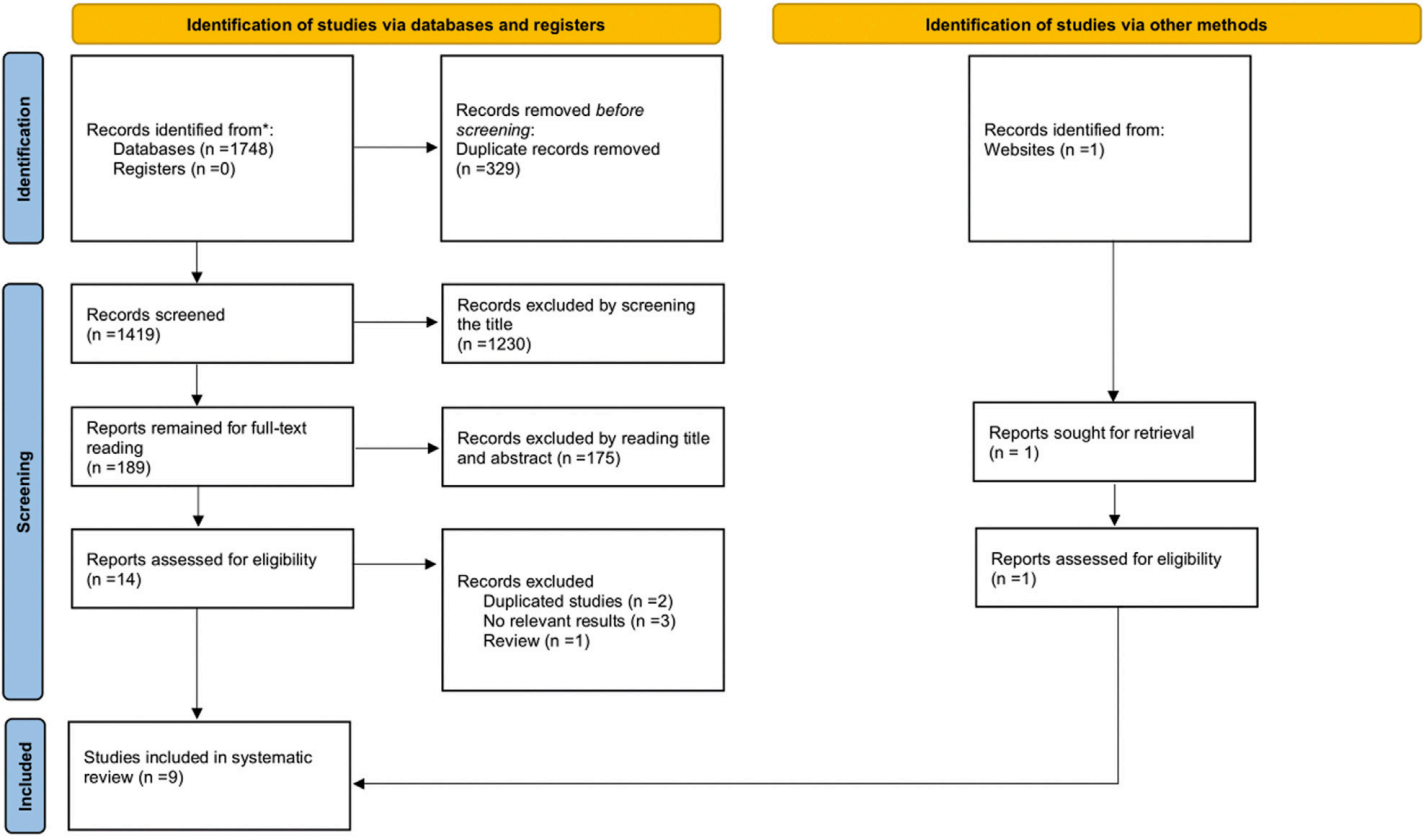
Flowchart of study selection based on PRISMA guidelines.

The search yielded a total of 1748 articles. After excluding 1734 articles, the remaining 14 articles were evaluated for eligibility. In addition, a keyword search on Google Scholar was conducted to obtain 1 eligible paper. We excluded one review, two duplicate cohort studies, and three studies that did not report a relevant outcome. Ultimately, we included nine studies (Hernigou and Deschamps, 2004a; Gulati et al., 2009; Boissonneault et al., 2013; Engh and Ammeen, 2014; Hamilton et al., 2016; Pegg et al., 2016; Liu et al., 2020; Kikuchi et al., 2021; Plancher et al., 2022). The search and screening process is detailed in the PRISMA flow diagram (Figure 1).

### Study characteristics

The nine included studies were all retrospective cohort studies two studies were conducted before 2010. The included studies were carried out in five countries across the three continents, including United States, the United Kingdom, France, China, and Japan. In these studies, one study did not report the gender proportion of participants, two studies had less than 50% female participants, and one study had more than 70% female participants. in reporting about UKA, three studies were conducted with less than 100 UKAs. Regarding the definition of ACL defect, one study did not report the definition of ACL, and one study found ACL defects in the post operative period. In terms of results reporting, eight studies reported outcomes related to postoperative revision, five studies reported Tegner activity scores, and four studies reported OKS. About Follow-up time, The mean follow-up time for the primary outcome of the studies was less than 10 years (Supplementary Tables S1, S2). In addition, all included studies were considered to be at low risk of bias based on NOS scores (Supplementary Appendix Table S3).

### Analysis of outcome measures

**FIGURE 2:**
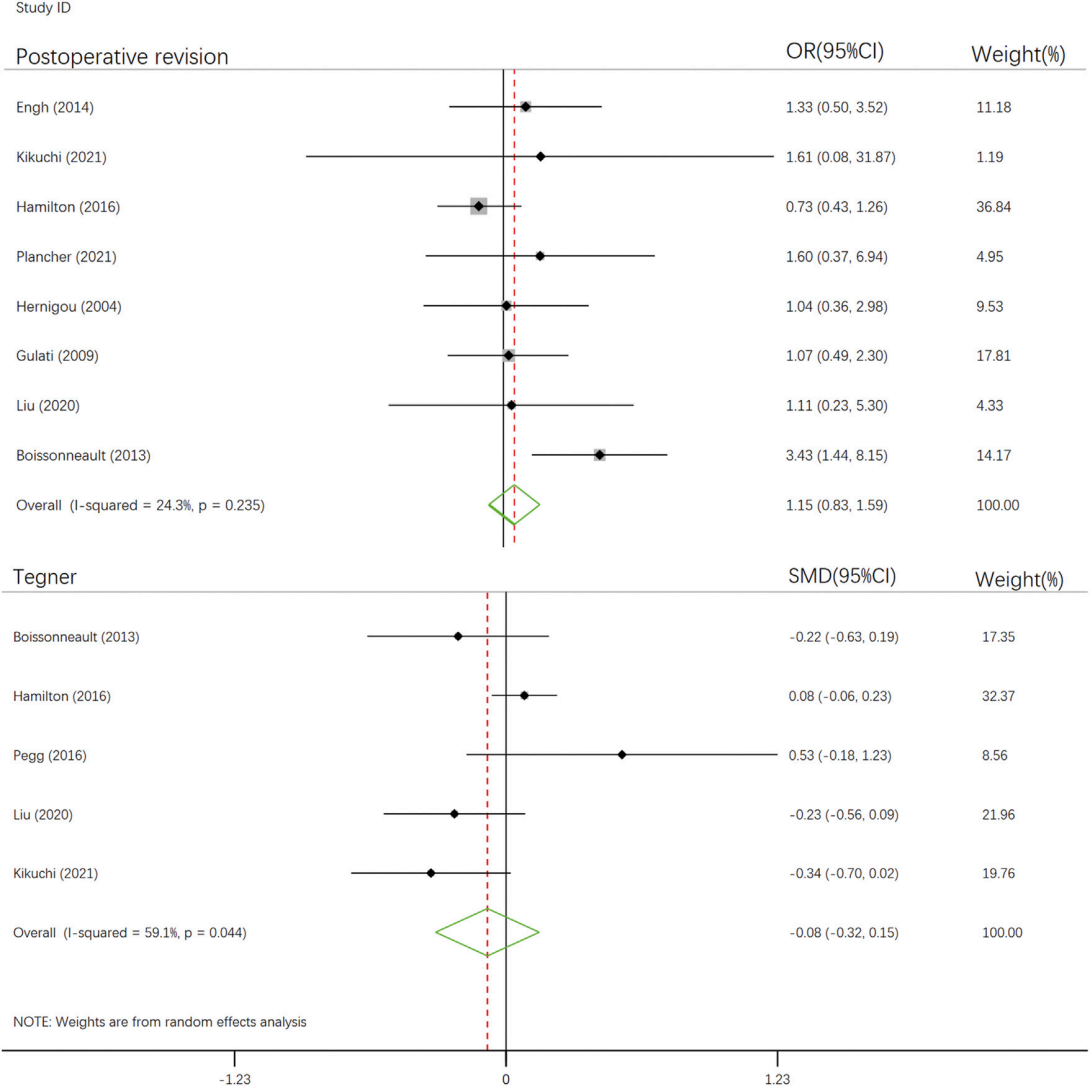
The forest plot for relationship between anterior cruciate ligament defect and postoperative outcomes after UKA.

Postoperative Revision (Hernigou and Deschamps, 2004a; Gulati et al., 2009; Boissonneault et al., 2013; Engh and Ammeen, 2014; Hamilton et al., 2016; Liu et al., 2020; Kikuchi et al., 2021; Plancher et al., 2022) was defined as having undergone a second surgery due to implant loosening or surgical failure. Outcomes associated with postoperative revision were reported in one article, of which two reported loosening of the implant, one reported postoperative posterolateral joint wear status, and six reported postoperative revision. There was no statistical difference in postoperative revision rates between intact and defective ACLs (OR, 1.15; 95% CI, 0.83–1.59; *p*-value, 0.235). Sensitivity analysis showed stability of this combined result by excluding studies before 2010 (OR, 1.19; 95% CI, 0.81–1.74; *p*-value, 0.101), two Asian studies (OR, 1.15; 95% CI, 0.82–1.60; *p*-value, 0.101), studies with less than 50% and more than 70% of women (OR, 0.94; 95% CI, 0.65–1.37; *p*-value, 0.737), studies with less than 100 UKAs (OR, 0.95; 95% CI, 0.65–1.38; *p*-value, 0.833), studies with age of participants more than 70 years old (OR, 1.16; 95% CI, 0.82–1.63; *p*-value, 0.407) and studies with follow-up less than 5 years (OR, 0.92; 95% CI, 0.61–1.39; *p*-value, 0.737). (Figure 2 and Supplementary Appendix Table S2).

Tegner activity score (Boissonneault et al., 2013; Hamilton et al., 2016; Pegg et al., 2016; Liu et al., 2020; Kikuchi et al., 2021) is a numerical scale (ranging from 0 to 10) used to assess work and physical activity levels (Tegner and Lysholm, 1985). Five articles reported on the Tegner activity score. According to the delineation and interpretation of SMD outcome indicators by Patrick Schober et al. (Andrade, 2020; Schober et al., 2021), there was no statistical difference in the postoperative follow-up Tegner activity score between ACL-intact and ACL-deficient group (SMD, -0.084; 95% CI, -0.320–0.151; *p*-value, 0.482). After excluding studies from Asia (SMD, 0.056; 95% CI, -0.222–0.334; *p*-value, 0.692), studies with lower 50 and higher 70 percentages of women (SMD, -0.042, 95% CI, -0.346–0.262; *p*-value, 0.786), studies with lower numbers of 100 UKAs (SMD, -0.127; 95% CI, -0.412–0.158; *p*-value, 0.382) and studies with follow-up longer than 10 years (SMD, -0.175; 95% CI, -0.435–0.084, *p*-value, 0.185), the result remained stable.

OKS (Boissonneault et al., 2013; Hamilton et al., 2016; Pegg et al., 2016; Kikuchi et al., 2021) is a patient-reported questionnaire that measures pain and daily activities (ranging from 0 to 48 points) (Dawson et al., 1998). Four articles reported OKS. Because of the paucity of reports on OKS and the possibility that direct merging of the data may yield unconvincing outcomes, here we only performed a qualitative systematic review of the results of the four studies rather than a quantitative synthetic analysis. Of the four studies, only one showed a moderate difference (SMD, -0.47; 95% CI, -1.17–0.24), and no significant effect of ACL deficiency on patients’ OKS scores after unicondylar knee arthroplasty was found in the remaining three studies. Considering the low sample size (2.38%) of the four studies included in this study combined, the confidence level of its findings is relatively low. The analysis of the above results shows that the defect of anterior cruciate ligament does not seem to be a risk factor for revision rate or functional recovery after UKA.

## Discussion

### Principal findings

This systematic review and meta-analysis showed that ACL defects and integrity do not affect the survival and functional scores of the prosthesis after UKA. This suggests that ACL defects are not a contraindication to UKA. The results of this study, which underwent systematic review of multiple outcome variables and Meta-analysis, showed no difference in postoperative revision rates, Tegner activity scores, and OKS between patients with ACL defects and intact patients undergoing UKA. The heterogeneity of the Meta-analysis results for postoperative revision (I^2^ = 24.3%) was low, suggesting a reliable result. In contrast, the Meta-analysis results of the Tegner activity score had significant heterogeneity (I^2^ = 59.1%), and the results need to be treated with caution. As for the OKS scores, only one of the included studies showed differences, but the results were not sufficiently convincing due to sample size limitations.

### Potential mechanisms

Common reasons for postoperative revision of UKA include progression of osteoarthritis, aseptic loosening, and bearing dislocation (Mohammad et al., 2018). During UKA, avoiding overfill is considered the most important factor in preventing the progression of osteoarthritis (Heyse et al., 2016). Studies on cadaver legs have also demonstrated that the overfilling causes a series of kinematic changes that can make knee valgus more severe and even lead to high strains of the medial collateral ligament (Heyse et al., 2016; Heyse et al., 2017). While overcorrection of valgus has also been shown to result in narrowing of the lateral joint space, causing an increased risk of UKA revision (Hernigou and Deschamps, 2004b; Khamaisy et al., 2016). A meta-analysis showed that Asian patients had a higher rate of revision after UKA compared to Western patients for a higher frequency of both deep knee flexion and internal femoral flexion, making the knee environment more susceptible to soft tissue imbalance (Ro et al., 2018).

Aseptic loosening is thought to be caused by small movements between the implant surface and the bone, which leads to fibrous membrane formation, trabecular microdamage and bone marrow edema, usually indicated by the presence of radiolucent lines on imaging (Fritz et al., 2015; Kleeblad et al., 2018a). A prospective study showed that the use of pulsed lavage significantly reduced the incidence of aseptic loosening, possibly due to deeper penetration of the bone cement into the lavaged cancellous bone, enhancing the strength of the cement interface and reducing micromovements (Clarius et al., 2009). This was also confirmed by 3D analysis and CT scans of the cemented implant interface in the cadaveric UKA tibia (Schlegel et al., 2011; Jaeger et al., 2013). Mechanisms of bearing dislocation include deep knee flexion, injury, and turning during sleep, and these events usually occur suddenly and are difficult for patients to detect (Fujii et al., 2015; Kawaguchi et al., 2019). As with aseptic loosening, the rate of dislocation is higher in Asian patients than in Western patients (Kim et al., 2014; Ro et al., 2018). We found that in studies conducted in Asia, the majority of those who underwent revision were due to loosening of the implanted prosthesis, whereas in study populations in Europe and the United States, the majority of patients who underwent revision were due to progression of knee osteoarthritis or trauma of unknown origin, which is consistent with previous findings.

We found only 23% of female patients in a cohort that showed significant difference in revision rates (Boissonneault et al., 2013). Another study had an only 11.1% percentage of female patients, showed significant difference in revision rates, but the same results did not appear in the Tegner score (Pegg et al., 2016). In addition, a systematic review and a joint national registry of arthroplasty showed a higher rate of revision of UKA in younger, more active people (Liddle et al., 2014; Kleeblad et al., 2018b). High levels of activity increase the risk of implant loosening due to wear and tear and may negatively impact revision rates (Naal et al., 2007) (63). Differences in exercise levels between patients of different ages and genders may be related to the heterogeneity of postoperative revision rates in UKA.

### Implications

Our meta-analysis provides an important reference for the choice of surgical approach for ACL-deficient patients with knee osteoarthritis. In the case of ACL deficiency, clinicians will likely be faced with three options, first to perform a single UKA, second to perform simultaneous ACL reconstruction and UKA, and last to perform TKA. In the case of ACL deficiency, our and other studies have shown that UKA and simultaneous ACL reconstruction are appropriate for active young adults, while isolate UKA is a reasonable option for older patients with reduced mobility (Mancuso et al., 2016; Ventura et al., 2017). The use of this procedure is still limited to elderly patients without knee instability. While fixed-bearing UKA compensates for the anterior-posterior stability of a single UKA in patients with ACL defects, rotational stability remains a prerequisite for its use (Zumbrunn et al., 2020). Some systematic reviews have shown that ACL reconstruction and UKA in ACL-deficient patients with knee instability and isolated medial septal pain are also beneficial in improving postoperative function and clinical outcomes (Volpin et al., 2018; Albo et al., 2021; El-Husseini et al., 2021). Due to the lack of reliable data to guide clinicians, physicians can consider the appropriate surgical option to use depending on the patient’s ACL injury, age and severity of knee osteoarthritis, etc.

### Research gaps

Compared to current studies, high-quality data from randomized controlled trials comparing ACL deficits with UKA outcomes are still lacking. More studies are needed to develop the assessment of other outcomes or scoring indicators such as postoperative recovery, OKS and so on. There are still some shortcomings in this study, including the lack of outcome indicators and sample size, as well as not covering patients of all ages. In this study, the average age of all the people involved is over 60 years old, but the incidence of ACL injury is very high in the process of teenagers’ sports, and the incidence of post traumatic arthritis (PTOA) is as high as 87% (Shelbourne and Stube, 1997), this also makes it impossible for this study to show whether ACL defects have an impact on young patients after single UKA. Besides, the long-term follow-up results of ACL reconstruction combined with UKA are still lacking (Tian et al., 2016; Legnani et al., 2021).

In future clinical research, we should recruit more patients, extend the follow-up time, define outcomes with more occurrences (including the use of compound outcomes), or combine the above methods to meet the sample size required to achieve the efficacy of the test. In addition, most of the studies included in this study were retrospective cohort studies with short-to medium-term follow-up, and longer follow-up or higher-level prospective studies are still needed to provide solid evidence-based medical evidence for the choice of clinical surgical modality. However, in future prospective studies, the problem of patients not wanting to undergo surgery or not wanting to risk conservative treatment may arise, which will cause some difficulties in the research (Smith et al., 2014).

## Conclusion

This systematic review and meta-analysis yielded no difference in the primary clinical outcome such as postoperative revision, Tegner activity score and OKS in ACL-deficient and UKA-intact patients. Thus, it is advisable for UKA to be performed in elderly patients with ACL deficiency. This would further expand the applicability of UKA and facilitate the reduction of patient injury, cost, and improvement of their quality of life compared to TKA.

## Data Availability

The original contributions presented in the study are included in the article/Supplementary Material, further inquiries can be directed to the corresponding authors.
