# Supplementary material for: Anterior cruciate ligament deficiency versus intactness for outcomes in patients after unicompartmental knee arthroplasty: a systematic review and meta-analysis
Source: Front Bioeng Biotechnol. 2022 Aug 23;10:890118. doi: 10.3389/fbioe.2022.890118 (PMC9445614; doi:10.3389/fbioe.2022.890118)
Supplement: Supplementary file 1 [file DataSheet3.docx]

**Appendix 3. Methodological quality score of the included studies based on the Newcastle–Ottawa scale (NOS) tool.**

| **First author** | **Year** | **Study Design** | **Selection** | | | | **Comparability** | **Exposure** | | | **Total Score** | **Risk of Bias** |
| --- | --- | --- | --- | --- | --- | --- | --- | --- | --- | --- | --- | --- |
|  |  |  | Representative­ness of cohort * | Selection of control cohort * | Ascertainment of exposure * | Outcome not present at start * | Comparability of cohorts ** | Assessment of outcome * | Length of follow-up * | Adequacy of follow-up * | **Total score 9*** |  |
| Hernigou | 2004 | Retrospective observational study |  | * | * | * | ** | * | * | * | 8 | low |
| Gulati | 2009 | Retrospective observational study |  | * | * | * | ** | * | * | * | 8 | low |
| Boissonneault | 2013 | Retrospective observational study |  | * | * | * | ** | * | * | * | 8 | low |
| Hamilton | 2016 | Prospective observational study |  | * | * | * | ** | * | * | * | 8 | low |
| Pegg | 2016 | Retrospective observational study |  | * | * | * | ** | * | * | * | 8 | low |
| Liu | 2020 | Retrospective observational study |  | * | * | * | ** | * | * | * | 8 | low |
| Kikuchi | 2021 | Retrospective observational study |  | * | * | * | ** | * | * | * | 8 | low |
| Plancher | 2022 | Retrospective observational study |  | * | * | * | ** | * | * | * | 8 | low |
| Engh | 2014 | Retrospective observational study |  | * | * | * | ** | * | * | * | 8 | Low |
