# Supplementary material for: Anterior cruciate ligament deficiency versus intactness for outcomes in patients after unicompartmental knee arthroplasty: a systematic review and meta-analysis
Source: Front Bioeng Biotechnol. 2022 Aug 23;10:890118. doi: 10.3389/fbioe.2022.890118 (PMC9445614; doi:10.3389/fbioe.2022.890118)
Supplement: Supplementary file 2 [file DataSheet1.doc]

**Appendix 1**

**Text S1 Search strategy**

**Database: PubMed from inception to Present> (Search date:** **Apr 19, 2022)**

--------------------------------------------------------------------------------

# Anterior Cruciate Ligament terms:

1. "Anterior Cruciate Ligament"[Mesh]
2. "Anterior Cruciate Ligament Injuries"[Mesh]
3. (Cruciate Ligament*, Anterior OR Anterior Cruciate Ligaments OR Ligament*, Anterior Cruciate OR Anterior Cranial Cruciate Ligament OR Cranial Cruciate Ligament* OR Cruciate Ligament*, Cranial OR Ligament*, Cranial Cruciate OR ACL OR anterior cruciate ligament status OR anterior cruciate ligament absent OR anterior cruciate ligament deficiency OR Anterior Cruciate Ligament Injur* OR ACL injur* OR Injur*, ACL OR Anterior Cruciate Ligament Tear* OR ACL Tear* OR Tear*, ACL)[Title/Abstract]
4. 1-3/or

**Arthroplasty, Replacement, Knee terms:**

1. "Arthroplasty, Replacement, Knee"[Mesh]
2. (Arthroplasties, Replacement, Knee OR Arthroplast*, Knee Replacement OR Knee Replacement Arthroplast* OR Knee Arthroplasty OR Arthroplasty, Knee OR Replacement Arthroplast*, Knee OR Arthroplasty, Replacement, Partial Knee OR Unicompartmental Knee Arthroplasty OR Arthroplasty, Unicompartmental Knee OR Knee Arthroplasty, Unicompartmental OR Unicondylar Knee Arthroplasty OR Arthroplasty, Unicondylar Knee OR Knee Arthroplasty, Unicondylar OR Partial Knee Arthroplasty OR Arthroplasty, Partial Knee OR Knee Arthroplasty, Partial OR Unicondylar Knee Replacement OR Knee Replacement, Unicondylar OR Partial Knee Replacement OR Knee Replacement, Partial OR Unicompartmental Knee Replacement OR Knee Replacement, Unicompartmental OR UKA OR UKR OR UCR OR Unicondylar arthroplasty OR Unicondylar replacement)[Title/Abstract]
3. 5-6/or

**Final search results: Combining Anterior Cruciate Ligament and Arthroplasty, Replacement, Knee and Outcome:**

1. 4 and 7 (842)

**Text S2 Search strategy**

**Database: EMBASE(Search date: Apr 19, 2022)**

--------------------------------------------------------------------------------

# Anterior Cruciate Ligament terms:

1. 'anterior cruciate ligament'/exp
2. 'anterior cruciate ligament injury'/exp
3. ('cruciate ligament*, anterior':ab,ti OR 'anterior cruciate ligaments':ab,ti OR 'ligament*, anterior cruciate':ab,ti OR 'anterior cranial cruciate ligament':ab,ti OR 'cranial cruciate ligament*':ab,ti OR 'cruciate ligament*, cranial':ab,ti OR 'ligament*, cranial cruciate':ab,ti OR 'acl':ab,ti OR 'anterior cruciate ligament status':ab,ti OR 'anterior cruciate ligament absent':ab,ti OR 'anterior cruciate ligament deficiency':ab,ti OR 'anterior cruciate ligament injur*':ab,ti OR 'acl injur*':ab,ti OR 'injur*, acl':ab,ti OR 'anterior cruciate ligament tear*':ab,ti OR 'acl tear*':ab,ti OR 'tear*, acl':ab,ti) AND [embase]/lim
4. #1 OR #2 OR #3

**Arthroplasty, Replacement, Knee terms:**

1. 'knee replacement'/exp
2. ('arthroplasties, replacement, knee':ab,ti OR 'arthroplast*, knee replacement':ab,ti OR 'knee replacement arthroplast*':ab,ti OR 'knee arthroplasty':ab,ti OR 'arthroplasty, knee':ab,ti OR 'replacement arthroplast*, knee':ab,ti OR 'arthroplasty, replacement, partial knee':ab,ti OR 'unicompartmental knee arthroplasty':ab,ti OR 'arthroplasty, unicompartmental knee':ab,ti OR 'knee arthroplasty, unicompartmental':ab,ti OR 'unicondylar knee arthroplasty':ab,ti OR 'arthroplasty, unicondylar knee':ab,ti OR 'knee arthroplasty, unicondylar':ab,ti OR 'partial knee arthroplasty':ab,ti OR 'arthroplasty, partial knee':ab,ti OR 'knee arthroplasty, partial':ab,ti OR 'unicondylar knee replacement':ab,ti OR 'knee replacement, unicondylar':ab,ti OR 'partial knee replacement':ab,ti OR 'knee replacement, partial':ab,ti OR 'unicompartmental knee replacement':ab,ti OR 'knee replacement, unicompartmental':ab,ti OR 'uka':ab,ti OR 'ukr':ab,ti OR 'ucr':ab,ti OR 'unicondylar arthroplasty':ab,ti OR 'unicondylar replacement':ab,ti) AND [embase]/lim
3. #5 OR #6

**Final search results: Combining Sarcopenia and Spine surgery and Outcome:**

1. #4 AND #7 (738)

**Text S3 Search strategy**

**Database: Cochrane Library from inception to Present> (Search date: Apr 19, 2022)**

--------------------------------------------------------------------------------

**Anterior Cruciate Ligament terms:**

#1 MeSH descriptor: [Anterior Cruciate Ligament] explode all trees

#2 MeSH descriptor: [Anterior Cruciate Ligament Injuries] explode all trees

#3 (Cruciate Ligament*, Anterior OR Anterior Cruciate Ligaments OR Ligament*, Anterior Cruciate OR Anterior Cranial Cruciate Ligament OR Cranial Cruciate Ligament* OR Cruciate Ligament*, Cranial OR Ligament*, Cranial Cruciate OR ACL OR anterior cruciate ligament status OR anterior cruciate ligament absent OR anterior cruciate ligament deficiency OR Anterior Cruciate Ligament Injur* OR ACL injur* OR Injur*, ACL OR Anterior Cruciate Ligament Tear* OR ACL Tear* OR Tear*, ACL):ti,ab,kw (Word variations have been searched)

#4 #1 or #2 or #3

**Arthroplasty, Replacement, Knee terms:**

#5 MeSH descriptor: [Arthroplasty, Replacement, Knee] explode all trees 2787

#6 (Arthroplasties, Replacement, Knee OR Arthroplast*, Knee Replacement OR Knee Replacement Arthroplast* OR Knee Arthroplasty OR Arthroplasty, Knee OR Replacement Arthroplast*, Knee OR Arthroplasty, Replacement, Partial Knee OR Unicompartmental Knee Arthroplasty OR Arthroplasty, Unicompartmental Knee OR Knee Arthroplasty, Unicompartmental OR Unicondylar Knee Arthroplasty OR Arthroplasty, Unicondylar Knee OR Knee Arthroplasty, Unicondylar OR Partial Knee Arthroplasty OR Arthroplasty, Partial Knee OR Knee Arthroplasty, Partial OR Unicondylar Knee Replacement OR Knee Replacement, Unicondylar OR Partial Knee Replacement OR Knee Replacement, Partial OR Unicompartmental Knee Replacement OR Knee Replacement, Unicompartmental OR UKA OR UKR OR UCR OR Unicondylar arthroplasty OR Unicondylar replacement):ti,ab,kw (Word variations have been searched)

#7 #5 or #6

**Outcome terms:**

#8 #4 and #7 (168)
