# Supplementary material for: Anterior cruciate ligament deficiency versus intactness for outcomes in patients after unicompartmental knee arthroplasty: a systematic review and meta-analysis
Source: Front Bioeng Biotechnol. 2022 Aug 23;10:890118. doi: 10.3389/fbioe.2022.890118 (PMC9445614; doi:10.3389/fbioe.2022.890118)
Supplement: Supplementary file 3 [file DataSheet2.docx]

**Appendix 2. Sensitivity analyses**

| Variables | OR/SMD | 95%CI | p Value for association | I^2^ Value, % | p Value for heterogeneity | Studies, n |  |  |  |  |  |  |
| --- | --- | --- | --- | --- | --- | --- | --- | --- | --- | --- | --- | --- |
| **Postoperative revision** | | | | | | |  |  |  |  |  |  |
| After 2010 | 1.19 | 0.81-1.74 | 0.382 | 45.4 | 0.101 | 6 |  |  |  |  |  |  |
| Exclude Asia | 1.15 | 0.82-1.60 | 0.423 | 45.7 | 0.101 | 6 |  |  |  |  |  |  |
| 50%≤Female≤70% | 0.94 | 0.65-1.37 | 0.759 | - | 0.737 | 5 |  |  |  |  |  |  |
| Numbers of UKAs≥100 | 0.95 | 0.65-1.38 | 0.791 | - | 0.833 | 6 |  |  |  |  |  |  |
| Follow-up＞5 years | 0.92 | 0.61-1.39 | 0.699 | - | 0.737 | 5 |  |  |  |  |  |  |
| **Tegner activity score** |  |  |  |  |  |  |  |  |  |  |  |  |
| Exclude Asia | | | | | | | 0.056 | -0.222-0.334 | 0.692 | 42.9 | 0.173 | 3 |
| 50%≤Female≤70% | -0.042 | -0.346-0.262 | 0.786 | 67.8 | 0.078 | 2 |  |  |  |  |  |  |
| Numbers of UKAs≥100 | -0.127 | -0.412-0.158 | 0.382 | 69.7 | 0.037 | 3 |  |  |  |  |  |  |
| Follow-up≤10 years | -0.175 | -0.435-0.084 | 0.185 | 36.2 | 0.195 | 4 |  |  |  |  |  |  |
| **Oxford Knee Score** |  |  |  |  |  |  |  |  |  |  |  |  |
| Exclude Asia | | | | | | | -0.051 | -0.187-0.086 | 0.468 | - | 0.446 | 3 |
| 50%≤Female≤70% | -0.467 | -1.170-0.236 | 0.193 | - | - | 1 |  |  |  |  |  |  |
| Numbers of UKAs≥100 | -0.228 | -0.550-0.093 | 0.164 | - | 0.455 | 2 |  |  |  |  |  |  |
| Follow-up≤10 years | -0.189 | -0.443-0.064 | 0.143 | - | 0.703 | 3 |  |  |  |  |  |  |

unicompartmental/unicondylar knee arthroplasty, UKA; anterior cruciate ligament, ACL; odds ratio, OR; standard mean difference, SMD; confidence interval, CI
